# Analysis of permanent second molar development in children born with cleft lip and palate

**DOI:** 10.1590/1678-7757-2019-0628

**Published:** 2020-06-08

**Authors:** Manuella Santos Carneiro ALMEIDA, Rosa Helena Wanderley LACERDA, Karolline Batista LEAL, Camila Helena Machado da Costa FIGUEIREDO, Bianca Marques SANTIAGO, Alexandre Rezende VIEIRA

**Affiliations:** 1 Universidade Federal da Paraíba João PessoaParaíba Brasil Universidade Federal da Paraíba , João Pessoa , Paraíba , Brasil .; 2 Universidade Federal de Campina Grande PatosParaíba Brasil Universidade Federal de Campina Grande , Patos , Paraíba , Brasil .; 3 University of Pittsburgh PittsburghPennsylvania United States of America University of Pittsburgh , Pittsburgh , Pennsylvania , United States of America .

**Keywords:** Cleft lip, Cleft palate, Dental Care, Diagnosis

## Abstract

The study of dental development in individuals born with cleft lip and palate (CLP) serves to determine when orthodontic intervention should start.

**Objective:**

To evaluate the permanent second molar development in children born with cleft lip and palate according to Demirjian’s and Nolla’s methods.

**Methodology:**

Out of a total of 513 digital panoramic radiographs, 113 pairs of children aged 3 to 16 years were selected. The exams were from children born with or without cleft lip and palate, of the same sex, with an age difference of up to 30 days. The images were analyzed by three examiners and reliability was checked through intra-examiner agreement by the Kappa test. The data were analyzed by Wilcoxon's and Mann-Whitney tests according to each dataset.

**Results:**

The findings indicated delayed development of the permanent second molars in children with CLP (P<0.001). The development of the right permanent second molar was delayed compared to the left molar in children with CLP. Moreover, mandibular teeth showed significantly earlier development than maxillary teeth in both the case and control groups. There was no significant difference in the development of permanent second molars between sexes.

**Conclusion:**

Children with CLP presented delay in the development of permanent second molars.

## Introduction

Oral clefts are congenital malformations resulting from the non-closure of the frontonasal and maxillary processes during the first weeks of embryonic life. These conditions may manifest as an isolated phenomenon or in association with other congenital anomalies. ^1 , 2^ Cleft lip associated or not with cleft palate, and cleft palate alone, are among the most common congenital malformations worldwide, affecting 1 in 700 newborns. ^3 - 6^ These birth defects have been attributed to genetic and environmental factors. ^7^

Individuals with cleft lip and/or palate (CLP) experience significant impact in their quality of life. ^8 , 9^ Morphological alterations resulting from these conditions may cause functional and esthetic issues, which, most often, lead to psychosocial distress. ^10^ The extension and severity of the cleft will determine the nature of the issues, which generally include difficulty in breastfeeding, recurrent infections of the respiratory tract and middle ear, hearing and speech alterations, as well as occlusal and facial aesthetic problems. ^10 , 11^

Orthodontic interventions are required for the rehabilitation of individuals with CLP. ^12^ Nevertheless, very early orthodontic interventions should be avoided due to poor stability, which makes the rehabilitation process even more exhaustive for patients and their families. ^10^ Several currently available methods can be used to determine the patient’s age or maturity of the dentition, most frequently Nolla’s ^13^ (1960) and Demirjian’s methods. ^14 - 16^

Demirjian’s method ^14^ categorizes dental development into eight stages (A to H), as follows: A - early calcification in the upper portion of the crypt, with cone or inverted cone shape and no fused calcification points; B - fusion of calcification points, cusp formation, occlusal surface delimitation; C - complete formation of the occlusal enamel, beginning of cervical extension, dentin deposition onto the upper portion, and early development of the pulp chamber contour; D - almost complete crown before the cemento–enamel junction is formed, well-defined pulp chamber ceiling; E - pulp chamber walls are better defined, root size smaller than crown height in posterior teeth, presence of pulp horns and onset of root bifurcation; F - pulp chamber walls forming an isosceles triangle, root size equal to or slightly larger than crown height; semilunar calcification in the furcation region of posterior teeth; wide conduits with beveled walls; G - parallel canal walls and partially open apex; H - apex closure.

Alternatively, Nolla ^13^ (1960) proposed a dental development classification which includes 11 stages, namely: Stage 0: absence of crypt; Stage 1: formed crypt; Stage 2: Early mineralization; Stage 3: 1/3 formed crown; Stage 4: 2/3 crown mineralization; Stage 5: almost complete crown formation; Stage 6: fully mineralized crown; Stage 7: 1/3 formed root; Stage 8: 2/3 formed root; Stage 9: root almost formed, and open apex; Stage 10: formed root and closed apex.

The individual’s chronological age is not always proportionally related to their stage of tooth development. ^17^ Hence, we hypothesized that such differences may be even more apparent among children with CLP.

Some studies have evaluated the dental age of individuals with CLP, ^12 , 18 - 23^ but these studies do not focus on differences in side despite cleft lip and palate affecting the left lip two out of three instances. Our objective was to investigate the dental age of children born with CLP using Demirjian’s and Nolla’s methods, ^13 , 14^ focusing on potential differences between cleft and non-cleft sides. Variations in tooth development between opposite sides in the dental arch have been reported in the literature. ^24 - 26^ Thus, our study hypothesis was that there are significant arch side-related differences in tooth development in this group of patients.

## Methodology

Five hundred and thirteen children treated at Cleft Lip and palate Center of Lauro Wanderley University Hospital (HULW), Federal University of Paraíba (UFPB), participated in this study. The inclusion criteria consisted of (i) children with CLP, (ii) aged 3 to 16 years, (iii) without any syndromes, systemic diseases or other orofacial clefts; (iv) of both sexes; (v) who had all permanent second molars (case group).

The sample size of the case group consisted of 113 digital panoramic radiographs. The distribution of cleft types is shown in Table 1 . In addition, a total of 113 radiographs were selected for the control group from children without CLP with characteristics matching those of the case group regarding age (difference of up to 30 days) and sex. Children in the control group were excluded if they had any documented syndromes or systemic conditions. Participants who did not meet the eligibility criteria were excluded from the analysis.

**Table 1:** Analysis of cleft types according to sex

	Sex				
Cleft Type	Boys		Girls		Total
	Right	Left	Right	Left	N
UCLP ^1^	8	21	7	25	61
UCL ^2^	-	7	1	3	11
BCLP ^3^	21		15		36
BCL ^4^	-		-		-
CP ^5^	-		5		5
TOTAL	57		56		113

1- Unilateral cleft lip and palate

2- Unilateral cleft lip

3- Bilateral cleft lip and palate

4- Bilateral cleft lip

5- Cleft palate

The sample was composed of 56 (49.6%) females and 57 (50.4%) males. This study was previously approved by the Institutional Review Board under protocol CAAE 23683913.0.0000.5181.

Three previously calibrated examiners (M.S.C.A; R.H.W.L; C.H.M.C) carried out the analysis. Twenty panoramic radiographs were used for calibration, and as such were not included in the final sample. Calibration lasted two weeks and intra-examiner agreement was measured by the weighted Kappa coefficient (pondered Kappa>0.91).

Patient information was concealed from the panoramic radiographs to prevent bias. The calibrated examiners evaluated the radiographic images simultaneously for each method, and the stages of development of the second molars were chosen by consensus. The evaluations were carried out in a darkened room on a 23-inch screen using Windows Image Viewer and Fax Program ^®^ (Windows XP).

Each second molar (Upper right permanent second molar - UR7, Upper left permanent second molar - UL7, Lower left permanent second molar - LL7 and Lower right permanent second molar - LR7) was classified according to the stages proposed by Demirjian’s and Nolla’s methods. ^13 , 14^

The stages of development of Demirjian’s method ^14^ were codified as follows: 1 for stage A, 2 for stage B, 3 for stage C, and so forth. Wilcoxon’s test was used for inter-group comparisons. The differences between the groups (case and control) for the same method, between the arch sides for the same group, and between the methods, correspond to paired data, which justifies the use of the non-parametric paired Wilcoxon’s test. Mann-Whitney’s test, which is not a paired test, was used for comparison between sexes. The data were statistically analyzed in SPSS version 21.0 (Statistical Package for the Social Sciences) with a 5% margin of error.

## Results

As shown in Table 2 , the analysis of second molars by Demirjian’s and Nolla’s methods revealed higher percentages of advanced stages of development (F, G, H for Demirjian’s method and 8, 9 and 10 for Nolla’s method) among individuals without CLP ( *P* <0.001). Moreover, Table 3 shows differences in the development of each permanent second molar between individuals with and without CLP and tooth development was found to be more delayed among individuals with CLP (case group), regardless of the tooth ( *P* <0.05).

**Table 2:** Analysis of the development stages of permanent second molars according to Demirjian’s and Nolla’s methods using Wilcoxon’s test for paired data

	Development			Development			
Tooth	stage	With cleft lip and palate	Without cleft lip and palate	stage	With cleft lip and palate	Without cleft lip and palate	P-value
	Nolla	n (%)	n (%)	Demirjian	n (%)	n (%)	
	1	-	-	A	6 (1.3)	1 (0.2)	p ^(1)^ < 0.001*
(UR7,UL7, LL7, LR7)	2	6 (1.3)	1 (0.2)	B	2 (0.4)	7 (1.5)	
	3	2 (0.4)	7 (1.5)	C	38 (8.4)	9 (2.0)	
	4	33 (7.3)	9 (2.0)	D	98 (21.7)	49 (10.8)	
	5	44 (9.7)	19 (4.2)	E	140 (31.0)	134 (29.6)	
	6	73 (16.2)	48 (10.6)	F	93 (20.6)	120 (26.5)	
	7	126 (27.9)	116 (25.7)	G	37 (8.2)	57 (12.6)	
	8	93 (20.6)	120 (26.5)	H	38 (8.4)	75 (16.6)	
	9	37 (8.2)	57 (12.6)				
	10	38 (8.4)	75 (16.6)				
	**TOTAL**	**452 (100)**	**452 (100)**		**452 (100)**	**452 (100)**	

(*): Significant difference at 5.0%.

(†): By the Wilcoxon's test for paired data.

**Table 3:** Analysis of the development stages of permanent second molars in individuals with or without cleft lip and palate, according to the classification method, using Wilcoxon’s test for paired data

		Group		
Tooth	Development	With cleft lip and palate	Without cleft lip and palate	*P-* value
	method	Median (P25;P75)	Median (P25;P75)	
UR7	Demirjian	5.00 (4.00; 5.00)	6.00 (5.00; 7.00)	p ^(1)^ < 0.001*
	Nolla	7.00 (6.00; 8.00)	8.00 (7.00; 9.00)	p ^(1)^ < 0.001*
UL7	Demirjian	5.00 (4.00; 6.00)	6.00 (5.00; 7.00)	p ^(1)^ < 0.001*
	Nolla	7.00 (6.00; 8.00)	8.00 (7.00; 9.00)	p ^(1)^ < 0.001*
LL7	Demirjian	5.00 (4.00; 6.00)	6.00 (5.00; 7.00)	p ^(1)^ < 0.001*
	Nolla	7.00 (6.00; 8.00)	8.00 (7.00; 9.00)	p ^(1)^ < 0.001*
LR7	Demirjian	5.00 (4.00; 6.00)	6.00 (5.00; 7.00)	p ^(1)^ < 0.001*
	Nolla	6.00 (7.00; 8.00)	8.00 (7.00; 9.00)	p ^(1)^ < 0.001*

(*): Significant difference at 5.0%.

(†): By Wilcoxon’s test for paired data.

Out of a total of 113 individuals with CLP, 13 showed different stages of development between the left and right arch sides, regardless of the cleft side. A relationship between the type and side of the cleft and the occurrence of delayed development of the second molars was investigated here. In individuals with left-UCLP, there was a greater frequency of delayed development on the right side, that is, on the non-cleft side (Fisher’s Exact test, *P* =0.05). As for those with right-UCLP, only one case showed delayed molar development, which occurred on the cleft side.

The frequency of delay in second molar development among individuals with bilateral clefts was compared against that of the control group. The findings indicated that bilateral clefts were significantly associated with delayed second molar development (Fisher’s Exact test, *P=* 0.01). Six out of 36 BCLP cases showed delayed development of at least one second molar, while only 5 out of 113 control cases showed delayed second molar development. No significant arch side–related differences in second molar development were observed.

As seen in Table 4 , Demirjian’s method revealed significant differences in the development of maxillary and mandibular second molars, with correspondingly greater development in the mandibular ones ( *P* <0.05). Demirjian´s (but not Nolla’s) method revealed differences between maxillary and mandibular tooth development in both the case and control groups.

**Table 4:** Analysis of the development stages of permanent second molars in individuals with or without cleft lip and palate, according to arch (maxilla or mandible), using Wilcoxon’s test for paired data

		Group		
Group of teeth	Method/Arch	With cleft lip and palate	Without cleft lip and palate	*P-* value
		Median (P25;P75)	Median (P25;P75)	
Second molars	**Demirjian**			
	Upper	5.00 (4.00; 6.00)	6.00 (5.00; 7.00)	p ^(1)^ < 0.001*
	Lower	5.00 (4.00; 6.00)	6.00 (5.00; 7.00)	p ^(1)^ < 0.001*
	*P-* value	p ^(2)^ =0.008*	p ^(2)^ =0.005*	
	**Nolla**			
	Upper	7.00 (6.00;8.00)	8.00 (7.00; 9.00)	p ^(1)^ < 0.001*
	Lower	7.00 (6.00;8.00)	8.00 (7.00; 9.00)	p ^(1)^ < 0.001*
	*P-* value	p ^(2)^ =0.262	p ^(2)^ =0.052	

(*): Significant difference at 5.0%.

(†): Using Wilcoxon’s test for paired data for the comparison between the second molar teeth in each arch.

(‡): Using Wilcoxon’s test for paired data for the comparison between arches.

Analysis of permanent second molar development in children born with cleft lip and palate

Table 5 shows no significant differences in the development of permanent second molars between sexes ( *P* >0.05).

**Table 5:** Analysis of permanent second molar development according to sex using the Mann-Whitney's test

Sex	Dermijian method		Nolla method	
	With cleft lip and palate	Without cleft lip and palate	With cleft lip and palate	Without cleft lip and palate
	Median (P25; P75)	Median (P25; P75)	Median (P25; P75)	Median (P25; P75)
Male	5.00 (4.00; 6.00)	6.00 (5.00; 6.00)	7.00 (6.00; 8.00)	8.00 (7.00; 8.00)
Female	5.00 (4.00; 6.00)	6.00 (5.00; 7.00)	7.00 (6.00; 8.00)	8.00 (7.00; 9.00)
Value of p	P ^(1)^ =0.396	P ^(1)^ =0.091	P ^(1)^ =0.168	P ^(1)^ =0.087

(*): Significant difference at 5.0%.

(1): Using Mann-Whitney’s test for the comparison between sexes in each group.

## Discussion

Demirjian’s method ^14^ categorizes dental development into eight stages, whereas Nolla’s method ^13^ includes 11 stages of development. As these methods use different analytical scores, it is impracticable to make a direct, inferential comparison between them other than a descriptive analysis. The advantage of using internationally validated methods is the possibility of comparing the findings across different populations and ethnicities. Demirjian’s and Nolla’s methods ^13 , 14^ were chosen in our study for their broad applicability worldwide. ^12 , 15 , 16 , 18 - 24 , 26^

While children with CLP are known to have normal genetic potential for growth, ^27 , 28^ some studies have demonstrated that they may present a delay in dental maturation. ^19 , 20 , 24^ The development of permanent dentition takes longer in children with CLP than otherwise and may be increased depending on the severity of the cleft. ^22^ In addition, eruption of the permanent teeth is slower on the side of the cleft. ^29^ Consistent with this, both methods in our study demonstrated a delay in the development of the permanent second molars in individuals with CLP, regardless of the cleft side, compared to those without any clefts.

Nolla ^13^ (1960) and Dhanjal, et al. ^30^ (2006) reported no significant differences in tooth mineralization in patients without malformation in the maxillomandibular complex, regardless of the arch side. Ribeiro, et al. ^31^ (2002) and Pioto, Costa, Gomide ^32^ (2005) compared the dental development of the maxillary lateral incisor in the region of the cleft with the development of its counterpart tooth in individuals with unilateral CLP. The authors observed a delay in tooth development on the cleft side. In our study, significant differences between the right and left sides were observed regarding the development of the second molar. Individuals with left-UCLP showed a greater frequency of delayed tooth development on the right side, that is, on the non-cleft side. Evidence suggests that during craniofacial development, some genes expressed on the left side are absent on the right side, ^33^ which is likely not due to chance alone.

Dhanjal, et al. ^30^ (2006) and Orhan, et al. ^34^ (2007) reported that mandibular teeth were considerably more developed than maxillary ones, similarly to what was observed in our study when using Demirjian’s method. ^14^ In contrast, similar tooth development was observed between the maxillary and mandibular arches when Nolla’s ^13^ method was used. It is worth noting that both methods differ in terms of graphical illustrations and codes for differentiation of the tooth development stages, as Nolla’s method ^13^ considers 11 stages while Demirjian’s method ^14^ consists of 8 stages. Hence, the differences observed between methods may be explained by the larger number of stages of development considered by Nolla ^13^ (1960). It can be considered more effective in characterizing the stages of tooth development than Demirjian’s method ^14^ and thereby reduce sharp variations taking place in between each stage.

Both methods are based on the evaluation of the seven left mandibular permanent teeth for estimating dental age, except for the third molar. Originally, the stages of development in both methods should be converted into values whose sum would indicate the dental age according to tables developed by the authors. However, the analysis of the mandibular left hemiarch alone does not consider comprehensive patient information such as a general overview of the patient’s dental development in each arch side. Moreover, the analysis of a single mouth quadrant may overlook important parameters, for instance, tooth development in the other three quadrants, arch side-related differences in tooth development, as well as the association between delayed tooth development, the type of cleft, and the non-cleft side. Thus, in our study, we considered only the graphical illustrations and tooth development codes from Nolla’s and Demirjian’s methods ^13 , 14^ and analyzed the permanent second molars of each mouth quadrant.

Our findings showed no significant difference in tooth development between the sexes ( *P* =0.396, *P* =0.168 – case group; *P* =0.091, *P* =0.087 – control group for Demirjian and Nolla Method, respectively). These results disagree with previously published studies, ^35 - 38^ where females were found to have earlier tooth development than males. ^36 , 38^ Intriguingly, Soares, et al. ^35^ (2015) and Ribeiro, et al. ^37^ (2018) observed earlier apical closure in males.

Heterogeneous distribution of cleft types was present in our study sample, which included unilateral and bilateral CLP, unilateral cleft lip and cleft palate. Herein, there were no children with bilateral cleft lip in the sample, hence further research addressing tooth development should consider a broader spectrum of cleft types. Moreover, due to the limited number of individuals with CLP in the sample, subjects were not excluded based on ethnicity. As this was a matched-control study, any ethnic differences that could potentially induce bias were eliminated through the sample matching process.

The determination of the most suitable time to start treatment depends directly on the stage of dental maturity. Very early orthodontic interventions are discouraged because of the high probability of relapse in the long term. ^10^ Hence, establishing the treatment start date is a critical aspect of the rehabilitation process, as individuals with CLP experience a long and complex rehabilitation history.

Taken altogether, our study reinforces the importance of conducting orthodontic planning based on the individuality of each patient and their CLP characteristics. Choosing the most appropriate moment to intervene not only renders treatment more effective but also contributes to the social and psychological compliance of cleft patients. The management of patients with clefts should comprehend humanized contact with them and include therapeutic options that require less exposure to inappropriate or untimely procedures.

## Conclusions

The dental development of permanent second molars by Demirjian’s and Nolla’s methods ^13 , 14^ is delayed in children born with cleft lip and palate.

Arch side–related differences in tooth development were observed in individuals with unilateral left cleft lip and palate, with delay most often observed on the right side, that is, on the non-cleft side.

Demirjian’s (but not Nolla’s) method revealed differences between maxillary and mandibular tooth development in both the case and control groups, but no differences were observed between sexes.
